# Indole pyruvate decarboxylase gene regulates the auxin synthesis pathway in rice by interacting with the indole-3-acetic acid–amido synthetase gene, promoting root hair development under cadmium stress

**DOI:** 10.3389/fpls.2022.1023723

**Published:** 2022-10-21

**Authors:** Gulmeena Shah, Sajid Fiaz, Kotb A. Attia, Naeem Khan, Muhammad Jamil, Adeel Abbas, Seung Hwan Yang, Tu Jumin

**Affiliations:** ^1^ Institute of Crop Science, College of Agriculture and Biotechnology, Zhejiang University, Hangzhou, China; ^2^ Department of Plant Breeding and Genetics, The University of Haripur, Haripur, Pakistan; ^3^ Department of Biochemistry, College of Science, King Saud University, Riyadh, Saudi Arabia; ^4^ Department of Agronomy, Institute of Food and Agricultural Sciences, Florida University, Gainesville, FL, United States; ^5^ Department of Biotechnology and Genetic Engineering, Kohat University of Science and Technology, Kohat, Pakistan; ^6^ Institute of Environment and Ecology, School of Environment and Safety Engineering, Jiangsu University, Zhenjiang, China; ^7^ Department of Biotechnology, Chonnam National University, Yeosu, South Korea

**Keywords:** plant-microbe interaction, IpdC gene, auxin signaling pathway, cadmium toxicity, root hair development

## Abstract

This research focused on cadmium (Cd), which negatively affects plant growth and auxin hemostasis. In plants, many processes are indirectly controlled through the expression of certain genes due to the secretion of bacterial auxin, as indole-3-acetic acid (IAA) acts as a reciprocal signaling molecule in plant–microbe interaction. The aim of current studies was to investigate responsible genes in rice for plant–microbe interaction and lateral root development due to the involvement of several metabolic pathways. Studies revealed that *GH3-2* interacts with endogenous IAA in a homeostasis manner without directly providing IAA. In rice, indole-3-pyruvate decarboxylase (*IPDC*) transgenic lines showed a 40% increase in lateral roots. Auxin levels and YUCCA (auxin biosynthesis gene) expression were monitored in *osaux1* mutant lines inoculated with *Bacillus cereus* exposed to Cd. The results showed an increase in root hairs (RHs) and lateral root density, changes in auxin levels, and expression of the YUCCA gene. *B. cereus* normalizes the oxidative stress caused by Cd due to the accumulation of ${\text{O}}_{2}^{-}$ and H_2_O_2_ in *osaux1* mutant lines. Furthermore, the inoculation *of B. cereus* increases DR5:GUS expression, indicating that bacterial species have a positive role in auxin regulation. Thus, the current study suggests that *B. cereus* and *IPDC* transgenic lines increase the RH development in rice by interacting with IAA synthetase genes in the host plant, alleviating Cd toxicity and enhancing plant defense mechanisms.

## Introduction

Plant-promoting rhizobacteria (PGPR) are the microbes that act as an efficient source of nutrition and are present in the plants’ close vicinity, especially in the rhizosphere (Esitken et al., 2010; Malusá et al., 2012). Some of these microbes significantly influence root morphogenesis and architecture (Yu et al., 2016; Grover et al., 2021). *Azospirillum brasilense*, as a PGPR, triggers the proliferation of lateral roots rather than stimulation of lateral root growth under unfavorable conditions (Grover et al., 2021). Cadmium (Cd) alters many essential processes (photosynthetic activity, carbon fixation, transpiration, destruction of biomolecules and organelles) in the plant; averted growth and chlorosis are generic symptoms caused by Cd. Furthermore, Cd interferes with the transport and uptake of P, Ca, Mn, Mg, Zn, K, and Fe in the root, resulting in leaf chlorosis and inhibiting plant growth (Xu et al., 2017; Haider et al., 2021). In rice, overaccumulation of Cd in vacuoles moderately limits the translocation to roots and shoots (Tezuka et al., 2010; Miyadate et al., 2011). Based on our previous study on the pleiotropic effect of rhizospheric bacteria plant response to different bacterial classes, *Bacillus cereus* pathogenic bacteria as a model microbe of Cd toxicity alleviator were used to study the phenomena of lateral root development (Jan et al., 2019). The microbial community responsible for nitrogen fixation and production of phytohormones was found as a key factor for plant growth development by PGPR and for the development of proficient root architecture for enhanced nutrient and water uptake (Mia and Shamsuddin, 2010; Egorshina et al., 2012; Meldau et al., 2012; Rajendran et al., 2012; Yu et al., 2016).

Auxin moves within the plant through polar and nonpolar auxin transport (Kramer and Bennett, 2006) and includes influx transporters of the AUXIN1/LIKE AUX1 (AUX1/LAX) family (Swarup et al., 2008); AUX1 mediates the transport of IAA and influences lateral root development (Marchant et al., 2002). Several rhizospheric bacteria such as pathogenic bacteria, PGPR, *Bacillus* sp., *Enterobacter* sp., *Azospirillum* sp., *Rhizobium* sp., and *Pseudomonas* sp. (El-Hadi et al., 2009; Khan and Doty, 2009; Merzaeva and Shirokikh, 2010; Roy et al., 2010; Celloto et al., 2012; Xu et al., 2018) can produce IAA. In microorganisms, the majority of the phytohormone-producing bacteria have been found to produce IAA. Auxin production in microorganisms is not only limited to pathogenic PGPRs but also includes plant growth-promoting fungi and endophytic bacteria that produce IAA (García Sánchez, 2018; Vázquez-Chimalhua et al., 2021). A few species of rhizospheric bacteria are known to produce IAA and act as signaling molecules for communication between bacteria to coordinate their activities (Xu et al., 2018). Many plant processes are indirectly controlled through the expression of certain genes due to the secretion of bacterial auxin (Olatunji et al., 2017; Hsu et al., 2021). The biosynthesis of bacterial IAA helps to promote plant growth *via* the indole-3-pyruvate decarboxylase enzyme, encoded by the indole-3-pyruvate decarboxylase (IpdC) gene, catalyzes the conversion of indole-3-pyruvate (IPyA) to indole-3-acetaldehyde (IAAId) (Sgroy et al., 2009). The indole acetamide pathway is a key route for the production of IAA in phytopathogenic strains. Indole-3-acetamide synthesis from tryptophan is catalyzed by tryptophan-2-monooxygenase. In PGPR, IAA biosynthesis proceeds through intermediate indole-pyruvic acid (Won et al., 2011), while in tryptophan-dependent pathway, a precursor gene ipdC to IAAId is followed by IAA formation (Wang and Fu, 2011; Duca and Glick, 2020). The roots exudate tryptophan, which acts as a precursor for auxin production and influences IAA synthesis in the majority of IAA-producing PGPR (Merzaeva and Shirokikh, 2010).

Cd modifies auxin homeostasis by negatively regulating specific auxin-related genes, resulting in altered cell differentiation and inhibiting root growth (Ronzan et al., 2018). In plants, the phenomena of IAA production in roots and interaction with IAA-producing PGPRs are not clear at the molecular level due to the existence of different metabolic pathways and genes involved in the synthesis of IAA in plants and PGPRs, evoking the question of which genes are responsible and how they interact with each other for the development of IAA and enhancing lateral root development. The current work was designed to find the responsible genes in rice root and *B. cereus* and the interaction between them that influences the plant and lateral root development.

## Material and method

### Bacterial growth conditions

The *Bacillus cereus* strain was first cultured on nutrient agar medium by the spread plate method containing 1% tryptophan, 2% yeast extract, 1% NaCl, and 1.5% agar A (w/v) for 24 h at 28°C. For inoculum preparation, a single colony was picked and cultivated in a liquid medium. Bacteria were grown aerobically for 24 h on a rotary shaker (120 rpm) at 28°C to attain the bacterial exponential phase. At 3,000 rpm, centrifugation was done for 8 min at 25°C for pelleting bacterial cells, washed thrice, and further resuspended in a sterile liquid medium as reported in previous studies by Jan et al. (2019).

### Construction and transformation of IPDC gene

For the 35S:IPDC transgenes, the ORF of the indole-3-pyruvate *decarboxylase* gene from the *Bacillus cereus* strain ((*ATCC-*14579) NC_004722.1) was cloned along with the 35S promoter into pCAMBIA1300 using the primers listed (**Supplementary Table S1**). Agrobacterium strain EHA105 was used to introduce 35S:IPDC and was transformed into the Japonica cultivar Dongjin, as previously reported by Hiei et al. (1994).

### OsAUX1 mutant’s identification

The identification of three independent T-DNA integration sites of auxin mutants (*osaux1*, *osaux1*-2, and *osaux1*-3) was analyzed by comparison with the SIGnAL database at http://signal.salk.edu/cgi-bin/RiceGE database as described by Yu et al. (2015). OsAUX1-specific T-DNA border primers were designed and used to confirm the insertion.

### Plant growth conditions

WT/Dongjin, *osaux1-1*, *osaux1-2*, *osaux1-3* mutant, and *IPDC* transgenic lines were germinated in MS^1/2^ medium at pH 5.8–6.2, as previously described by Wang et al. (2014). The Cd treatment was performed by using 200 µM of Cd for 4 to 7 days; the duration of Cd treatments is indicated in the legends of figures for each experiment.

### RNA extraction and quantitative RT-PCR

Total RNA was extracted from roots using the Trizol plant RNA extracting method as described by Wang et al. (2010a). To analyze of the Aux1 expression of *osaux1* mutant, reverse transcriptase PCR (RT-PCR) was performed using the Takara kit (http://www.takara-bio.com) as per manufacturer’s instructions previously described by Wang et al. (2014). The primers used in this study are listed in **Supplementary Table S1**.

### Subcellular localization of OsAux1

35S:IPDC:sGFP and 35S:*OsGH3* fusion construct was transiently expressed by agrobacterium-mediated transformation in rice protoplast, as previously described by Yoo et al. (2007). Confocal microscopy (Olympus fluoview fv300) was used to acquire images.

### GUS staining and analysis of GUS activity

For GUS, staining wild-type and *osaux1* mutant seedlings transformed with *DR5:GUS* were primed with *B. cereus*, and GUS was performed as described by Ulmasov et al. (1997). After staining, roots were socked with 70% ethanol to remove the extra dye. Jefferson et al. (1987) method was used for the quantification of Gus activity by scrutinizing cleavage of 4-methylumbelliferyl-β-d-glucuronide (https://www.sangon.com). Canon EOS 800D was used to acquire GUS images.

### Analysis of auxin and tryptophan

For analysis of IAA concentration, 15 to 20 mg FW of 5-day-old seedling of wild-type and *osaux1* mutant inoculated with or without *B. cereus* was washed with deionized water three to five times and ground into a fine powder using liquid nitrogen. The extraction of free IAA was performed as described by Wang et al. (2014), while gas chromatography was performed for purification and quantification as described by Ljung et al. (2005). Salkowski reagent was used for the analysis of IAA content by bacterial strain, and *ipdc* mutant was performed as described by Sarwar et al. (1992). For measurement of tryptophan content, the free amino acid was extracted from 5-day-old seedlings of wild-type and *osaux1* mutant inoculated with or without *B. cereus* as described by Muller and Touraine (1992). Roots were kept in 80% ethanol for 12 h at 4°C, 1 h in 60% ethanol, and 24 h in deionized water at 4°C. The supernatant was fractioned and kept at −20°C, and tryptophan content was determined by using HPLC (Shimadzu, LC-20A series Japan).

### Cd localization and quantification

To visualize the distribution of Cd (200 µM) in the root zone of wild-type and *osaux1-3* mutant with or without *B. cereus* inoculation from 7-day-old seedlings was determined by using the method mentioned by Jan et al. (2019). Roots were first washed with deionized water and then with 0.01 µM of EDTA solution, dried at 80°C until the material reached constant weight. In total, 100 mg of root sample was digested with sulfuric acid and nitric acid mixture. Digested samples were diluted with 20 ml of deionized distilled water and filtered using Whatman filter paper. Cd content was measured using a Synergy H1 atomic absorption spectroscope. For Cd analysis in bacterial culture, *B. cereus* was grown in an LB medium. After 18 h, *B. cereus* was treated with 400 µl of Cdcl_2_ and grown for a further 4 h. The image was micro-graphed using the Nikon Eclipse DS-Ri2 microscope.

### Quantification of H_2_O_2_ content and cell death detection by Evans blue, Hoechst 33342

In roots, H_2_O_2_ levels were measured using the methodology described by Jana and Choudhuri (1982). The roots were homogenized in 6 ml of 50 mmol/l PBS (pH 6.5). The absorbance was read at 410 nm with a Synergy H1 atomic absorption spectroscope. The primary roots of 7-day-old seedlings treated with 200µM CdCl_2_ were excised from 20 seedlings of each wild-type and *osaux1* mutant with or without *B. cereus* inoculation and stained in 0.025% (w/v) Evans blue solution for 20 min at 25°C and washed with ddH_2_O to eradicate excess dye. Root tips were immersed in 1% SDS and 50% methanol for 1 h at 50°C. The absorbance was measured at 600 nm with a microtiter plate using the protocol of Baker and Mock (1994). To see the cell wall integrity between wild-type and mutant line, roots were stained with propidium iodide for 20 min and then washed three to five times with 0.85% NaCl and micrographed using confocal microscopy. To study the nucleus in interphase cells, roots were washed three times with PBS solution (pH 7.4) and immersed in Hoechst labeling solution (http://cshprotocols.cshlp.org/) 1:100 solution for 30min at 25°C. The labeling solution was aspirated from the roots, washed three times with PBS, and micrographed at 483 nm with a Nikon Eclipse DS Ri2 microscope.

## Results

### Characterization of AUX1 and loss of function lines

**Figure 1:**
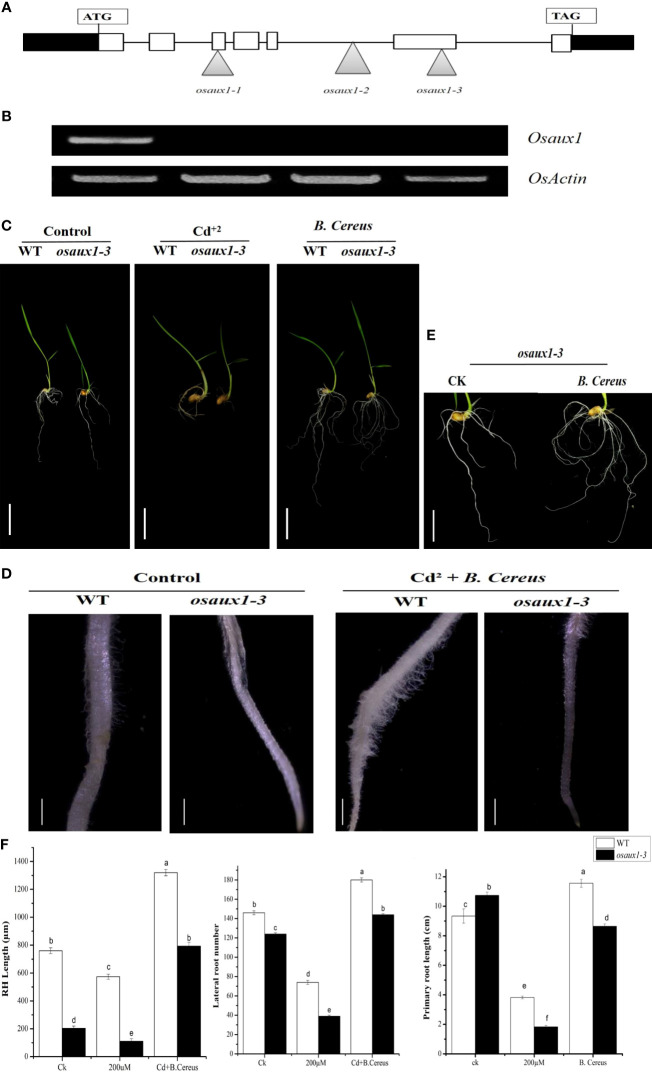
Three T-DNA insertion lines were identified in the *OsAux1* gene. **(A)** Schematic representation of T-DNA insertion mutations in *osaux1*-1, *osaux1*-2, and *osaux1*-*3* in the *OsAux1* gene. The open arrow heads represent T-DNA insertion positions. Filled white boxes represent exons; black boxes represent untranslated region; black lines represent introns. **(B)** Detection of RT-PCR of *OsAuxX1* transcripts in WT (Dongjin) *osaux1-1*, *osaux1-2*, and *osaux1-3*; actin transcripts were amplified as a control. **(C)** Seven-day phenotypic comparison of grown seedlings of wild-type (Dongjin), mutant lines *osaux1-3*, and **(B)** *cereus*-inoculated under Cd treatment. Scale bar, 1 cm. **(D)** Root hair morphology of **(B)** *cereus*-inoculated and noninoculated wild-type and *osaux1-3* after 3 days of treatment with 200 µM CdCl_2_ using Nikon AZ100. Scale bar, 1 mm. **(E)** Focused image of *osaux1-3* mutant under control. **(F)** Root phenotype quantification of PR length, LR root number, and RH length in wild-type and mutant lines grown in Cd bacterial-inoculated and noninoculated (controlled) conditions. Five seedlings from each of three replicates were assessed and grown for 10 days. Lowercase letters indicate significant differences between means, determined using Duncan’s multiple range mean comparisons.

The Salk database (http://signal.salk.edu/cgi-bin/RiceGE) led to the identification of three T-DNA integration sites for auxin mutant (*osaux1*-1, *osaux1-*2, and *osaux1-*3) in the Dongjin (DJ) background. A homozygous loss of function mutant was identified by PCR using DNA and RNA levels (**Figure 1A**). The RT-PCR analysis regarding auxin mutants analogous to the insertion sites did not detect any transcript of the *osaux1-3* mutant (**Figure 1B**). To study the role of *B. cereus* on root architecture of lateral root development, 5-day-old germinated wild-type and *osaux1* mutants inoculated with and without bacterial strain were transferred to fresh agar medium for an additional 4 days in the presence of Cd at 200 µM (**Figures 1C–E**). Under noninoculated conditions, auxin mutants exhibit lowered primary root length and root count as compared to wild-type seedlings, which are dramatically reduced in Cd-treated seedlings (**Figure 1F**). The inoculation with *B. cereus* amended the number of lateral roots in wild-type and mutant plants, and the density of lateral roots (LR) became higher in both auxin mutant and wild-type under Cd exposure (**Figure 1C**), confirming that *B. cereus* had a positive role in indusial growth rate.

### OsAux1 expression in the PRs, LRs, and RHs of rice

**Figure 2:**
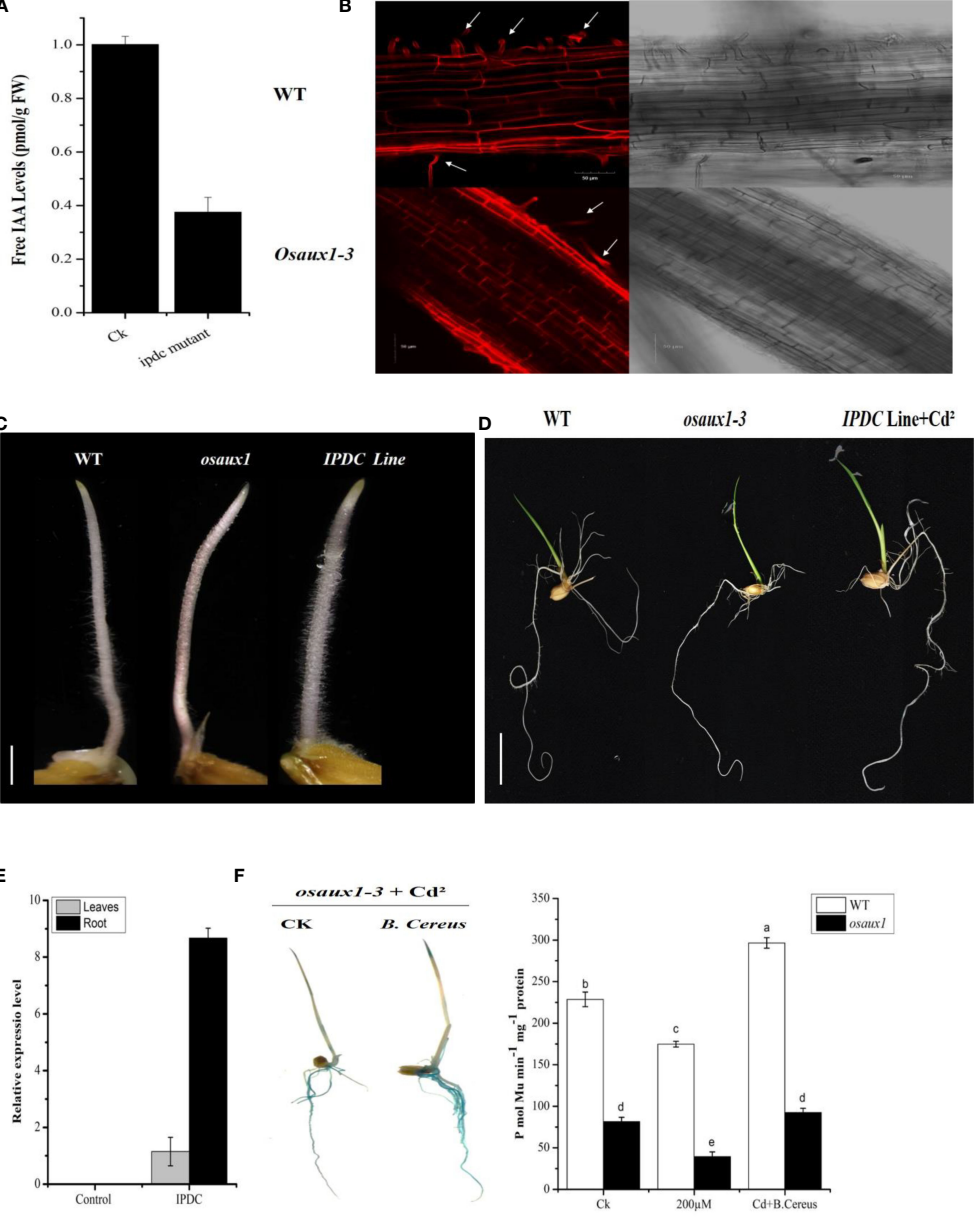
**(A)** Expression of indole-3-acetic acid by **(B)** *cereus* and an *ipdc* mutant. **(B)** Confocal micrographs showing propidium iodide staining of the cell wall of wild-type and *osaux1-3* mutant from the lateral root tips of *Oryza sativa*. L. Micrographic representation of cell size of the wild-type (above row) and *osaux1-3* mutant (below row). Arrow indicates the RHS development. Right, the bright field with a scale bar of 50 µm. **(C, D)** Phenotypic comparison between wild-type (Dongjin), mutant lines *Osaux1-3*, and *IPDC transgenic line* after 3 to 7 days of germination. Scale bar, 1 cm. **(E)** Relative expression of IPDC transgenic lines in root and shoot. Scale bar, 1 cm. **(F)** *osaux1-3* inoculated and noninoculated with **(B)** *cereus* expression in 10-day-old seedlings treated with 200 µM CdCl_2_; DR5*:GUS* was transformed into the rice. Ten positive transgenic lines were observed. *ProDR5*:GUS activity is shown in **(F)**. Lowercase letters indicate significant differences between means, determined using Duncan’s multiple range mean comparisons.

To understand the molecular mechanism of plant–microbe interaction, transgenic lines of IPDC and *osaux1-1* mutant were visualized phenotypically or morphologically. The expression pattern of free IAA in the *ipdc* mutant line shows a strong reduction in IAA content (**Figure 2A**) in comparison to the control (*B. cereus*). From root surface examination, we found that the *Osaux1-3* mutant lines had shorter outer sheath or dermic cells in comparison to wild-type (**Figure 2B**). In response to Cd exposure, the LRs were much shorter as compared to wild-type, and the RH density decreased 35.5% in *Osaux1-3* but only 25.5% in wild-type. Interestingly, the RH density in IPDC transgenic lines was greater than 60% (**Figures 2C, D**), indicating that the auxin-dependent precursor of bacterial species influences the RH growth and density. In roots, IPDC transgenic line showed higher expression as compared to the shoots (**Figure 2E**).

### Auxin expression in root cell and localization in the nuclear membrane

**Figure 3:**
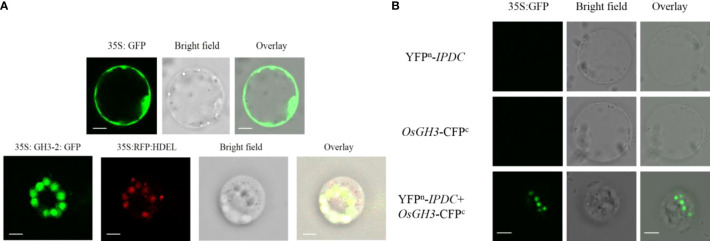
**(A)** Subcellular localization of 35S:*GH3-2*:GFP using rice protoplast 35S; green fluorescent protein (GFP) was used as a positive control. Left to right: green fluorescent protein of OsGH3-sGFP is shown in green color; red fluorescence shows RFP:HDEL (ER marker), bright field, and overlay microscope images. GH3s are most consistently localized to the ER membrane. Scale bars = 10 µm. **(B)** Protein interaction of *GH3* and *ipdc* using rice protoplasts. Scale bars = 10 µm.

To further investigate the auxin involvement in response to bacterial interaction, *osaux1-3* mutant negatively affects the LR development in response to Cd, which was promisingly upregulated in the inoculated mutant line (**Figure 1F**). By using the GUS assay, we examine the molecular mechanism of AUX1, which regulates RH development in rice. DR5:*GUS* staining in rice plants demonstrated that Cd reduced expression in PR, LR, and root hair in the *osaux1-3* mutant line, but increased dramatically in *B. cereus*-inoculated rice seedlings, indicating that *B. cereus* interacts in the rhizosphere and plays a positive role in auxin regulation (**Figure 2F**). To further understand the plant–microbe interaction of AUX1 and *B. cereus*, the colocalization of *OsGH3-2* revealed that GH3s are most consistently localized to the ER membrane by using RFP:HDEL (ER marker) (**Figure 3A**) in rice protoplast. Protein interaction was localized transiently in ER membrane with *YFP^n^-IPDC* and *OsGH3-*CFP^c^ expression vectors in the rice protoplast (**Figure 3B**), indicating that *Bacterial* spp. produce ipdC protein exogenously that further interacts with indole-3-acetic acid–amido synthetase gene protein (*GH3-2*) precursor and increases auxin content in roots, resulting in an increase in lateral roots and root hair.

### OsAux1 mutants are sensitive to Cd stress and induced cell death

**Figure 4:**
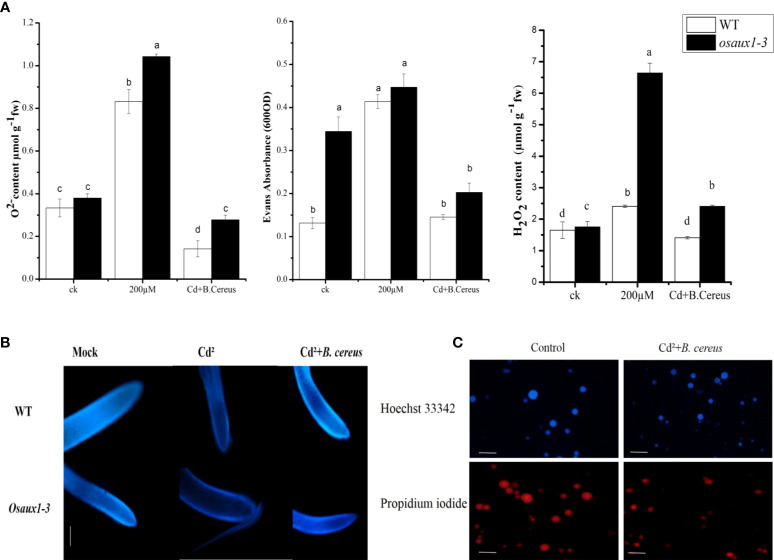
**(A)** Effect of Cd augmentation on H_2_O_2_, O_2_, and cell death in rice. Accumulation of ${\text{O}}_{2}^{-}$ and H_2_O_2_ was measured in wild-type and *osaux1-3* mutant exposed to 200 µM of CdCl_2_ for 7 days. Cell death was also assessed after days of wild-type and *osaux1-3* by Evans Blue staining exposed to 200 µM of CdCl_2_ for 7 days. **(B)** Histochemical detection of **(B)** *cereus*-inoculated and noninoculated wild-type and *osaux1-3* root tips exposed to 200 µM of CdCl_2_ with Hoechst 33342 staining image was visualized after 7 days of stress using Nikon Eclipse DS Ri2 microscope. Scale bars = 2µm. **(C)** Cultural staining of **(B)** *cereus with* Hoechst 33342 and propidium iodide in the presence of Cd. The bacterial culture was grown in an LB medium with 10^8^ CFU. Lowercase letters indicate significant differences between means, determined using Duncan’s multiple range mean comparisons.

H_2_O_2_, ${\text{O}}_{2}^{-}$ , and cell death were quantified to fully understand the augmented compassion towards the Os*AUX1* under Cd stress. As compared to wild-type, the amount of O_2_ produced by the mitochondrial electron transport chain was determined in *osaux1-3* mutants under Cd treatment. At 200 µM of Cd treatment, H_2_O_2_ content in the *osaux1-3* mutant was high as compared to wild-type, while it became considerably low in bacterial-inoculated seedlings (**Figure 4A**). Moreover, Hoechst 33342 staining was used to quantify the cell integrity in rice root tips. Results revealed that the root tips of *osaux1* mutant showed lower cell integrity as compared to wild-type in response to Cd stress, while in bacterial-treated *osaux1* mutants (**Figure 4B**), the accumulation of Hoechst 33342 staining was high as compared to noninoculated seedlings, thus indicating that in *osaux1* mutant, cell death is most likely caused by Cd stress. The death rate was also high in bacterial culture in response to 200 µM of Cd treatment with Hoechst 33342 and propidium iodide staining (**Figure 4C**).

### Metal absorption effect Cd, AUX1, and tryptophan content

**Figure 5:**
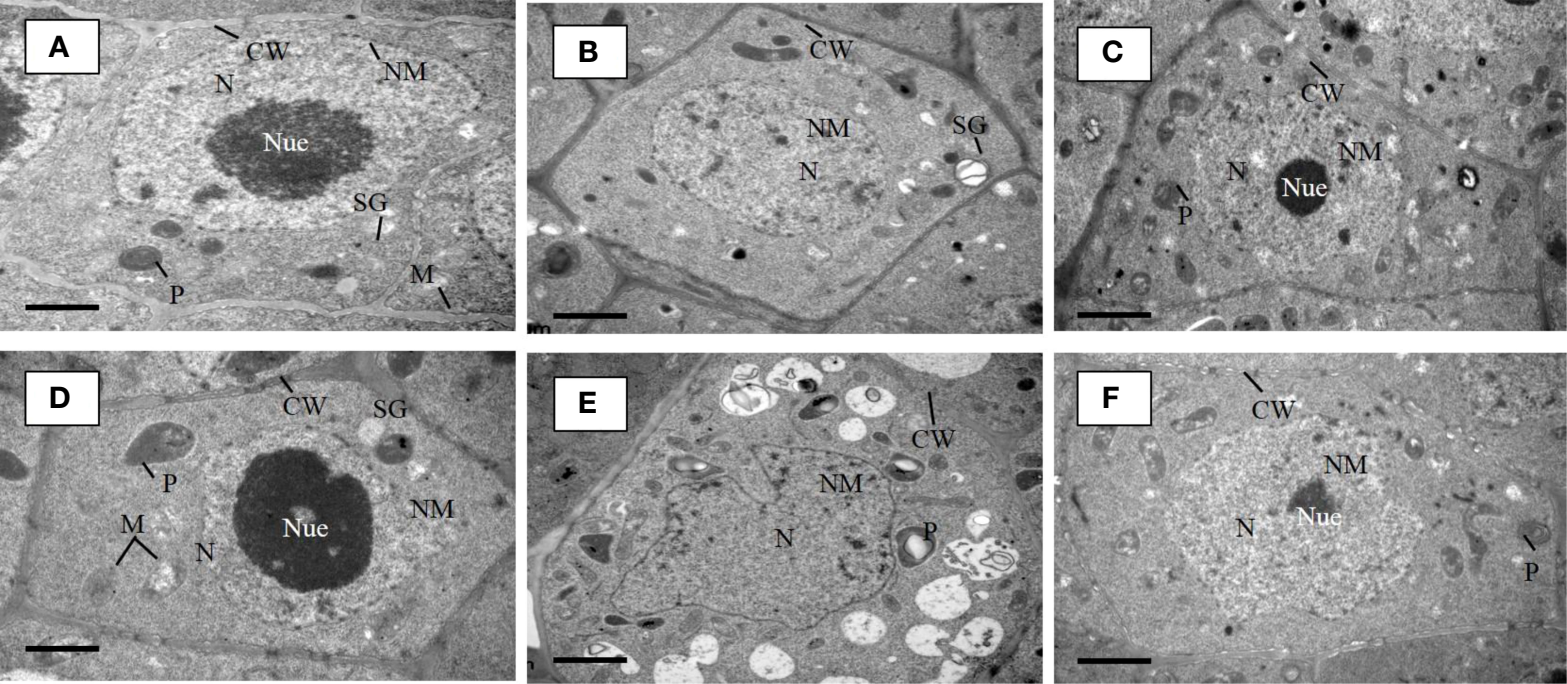
TEM micrographs of lateral root tip cells of 7-day wild-type **(A)** and *osaux1-3* **(D)** mutant under control condition, 200 µM CdCl_2_ **(B, E)**, and 200 µM CdCl_2_ with *B. cereus* inoculated **(C, F)**. **(A)** Root tip cells of Dongjin as a wild-type cultivar and **(D)** *osaux1-3* mutant under control **(CK)** show well-developed cell wall (CW), oval-shaped and clear mitochondria (M), starch grain (SG), plastids (P), nucleus (N), nuclear membrane (NM), and nucleolus (Nue). TEM micrographs of root tip cells of **(B)** wild-type and **(E)** *osaux1-3* under 200 µM Cd show immature nucleus (N), plastids (P), and scattered nucleolus (Nue) and a discontinue cell wall (CW). TEM micrographs of root tip cells of **(C)** wild-type and **(F)** *osaux1-3* under combined 200 µM Cd and bacterial treatment show well-developed cell wall (CW) and nucleus (N) along with a recovered nucleolus (Nue).

**Figure 6:**
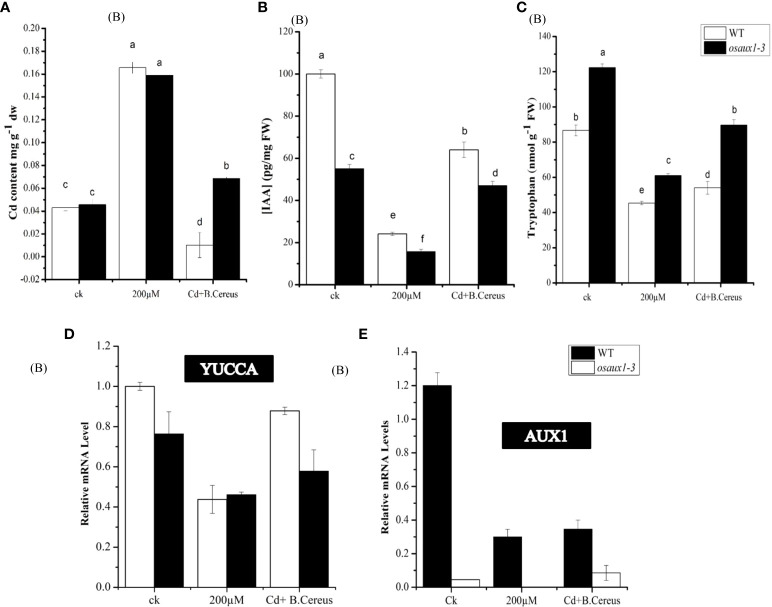
Effect of Cd on IAA and tryptophan content in wild-type and *osaux1-3* content. **(A)** Cadmium content was measured in root **(B)**, indole 3-acetic acid (IAA) **(C)**, and tryptophan content in WT and *osaux1-3*. Rice seedlings were inoculated with or without **(B)** *cereus* and 200 µM CdCl_2_ for 4 days in three biological repeats. **(D, E)** Relative mRNA levels of YUCCA and AUX1 in roots of wild-type and *osaux1-3* after 3 days of treatment with 200 µM CdCl_2_ and **(B)** *cereus*. Three biological repeats were included for quantitative RT-PCR analysis. Lowercase letters indicate significant differences between means, determined using Duncan’s multiple range mean comparisons.

We examined the Cd augmentation in root tips by transmission electron microscopy (TEM) (**Figure 5**) and by quantifying the Cd content in root tips (**Figure 6A**). The Cd content in the *osaux1* mutant was insignificantly high as compared to the wild-type as quantified by using the Synergy H1 spectroscope. The TEM qualification assay showed damaging results in *osaux1* mutants as compared to wild-type and *Bacillus*-inoculated root tips. The nucleolus gradually becomes invisible in Cd-treated seedlings of *osaux1* mutants and wild-type, indicating that Cd penetrates roots by cell rupturing (**Figure 5**). Tryptophan serves as a precursor for the biosynthesis of IAA. In the *osaux1* mutant, the free IAA content was less upon exposure to Cd (**Figure 6B**) whereas tryptophan content was higher (**Figure 6C**), while bacterial-inoculated seedlings resulted in normalizing the free IAA content in roots.

### Expression of biosynthesis and transport genes

Upon inoculation with *B. cereus*, the level of YUCCA and AUX1 triggered and showed upregulation in response to the Cd treatment in both wild-type and *osaux1* mutants (**Figures 6D, E**). Other genes such as TSA, TSB, and ASA involved in the biosynthesis of tryptophan, IAA, and glucosinolate synthesis were stimulated two- to threefold (data not shown). The overall picture indicated that *B. cereus* strongly influences the root IAA production.

## Discussion

In monocotyledonous species such as rice, lateral roots are essential organs that increase the absorbing capacity of water (Zhao et al., 2015). In early reports, *AUX1* and *LAX3* in *Arabidopsis* serve as LR and RH regulators (Jones et al., 2009). Biosynthetic signaling pathways get activated by auxins secreted by PGPR (Roy et al., 2010). Root morphology changes due to the disruption of the *AUX1* inhibited gene upon inoculation of rhizobacteria suggest that IAA is the main factor in plant–microbe interaction. Heavy metal uptake in roots mainly causes cell rupturing and infects plant integrity (Jan et al., 2019). When plants are exposed to high levels of toxic compounds, the roots are the primary part severely affected by such a level of abiotic stress. In our previous study, we found *B. cereus* is involved in plant growth development under Cd stress and acts as a PGPR (Jan et al., 2019). In the current study, we further investigated the involvement of auxin influx and signal transduction pathways in rice’s LR and RH development in response to *B. cereus*. In *Arabidopsis*, auxin is not only expressed in root hair but also regulates root hair development. *AUX1* has been reported as an essential expressing gene during lateral root primordia initiation (Yu et al., 2016). In recent years, scientists have started paying close attention to auxin signaling and heavy metal response (Tamás et al., 2015). In our study, the *osaux1* mutant showed a significant decrease in the lateral root number, and root hair indicated that *AUX1* plays a key role in lateral root development in rice. Interestingly, the inoculation with rhizospheric bacteria showed an increase in primary and lateral root density in *osaux1* mutant under control (Dongjin wild-type) and 200 µM of Cd treatment, suggesting that Aux1 might be able to control or activate lateral root formation due to IAA accumulation in roots, similar to the finding of Zhao (2010) and Casanova-Sáez et al. (2021). Different microbial pathways exist for IAA synthesis (Spaepen et al., 2007). The indole-3-pyruvate pathway is one of the predominant pathways for plant root development in rhizobacteria like *Azospirillum brasilense*. An increase in the level of exogenous IAA in the rhizosphere by bacterial strains showed similar results to IPDC transgenic lines, suggesting that ipdc as precursor gene of auxin biosynthesis decisively affects plant development. It is therefore conjectured that bacterial auxin produced by PGPR may amend the level of auxin and affect the auxin-regulated physiological processes. Similar findings were also reported by Jasim et al. (2014) and Jijón-Moreno et al. (2015) that ipdC encodes the PPDC gene, which is a key enzyme in producing IAA growth hormone. Furthermore, the rate-limiting step is catalyzed by ipdC, and the mutation of ipdC leads to a strong reduction of IAA, similar to the finding of Baudoin et al. (2010). In bacterial *ipdc* knockout mutant, the level of IAA was not significantly higher than that of *B. cereus*, demonstrating that the ipdc gene plays a role in both Trp-dependent and Trp-independent biosynthesis pathways, with an average of 61 nmol g^−1^ FW tryptophan measured in the Cd treatment *osaux1-3* mutant, which increased significantly with bacterial inoculation. Tryptophan serves as a precursor for the biosynthesis of IAA (Zhao et al., 2015). Results revealed that bacterial inoculation significantly increases the level and distribution of IAA in response to Cd in the roots of *osaux1-3* than that of noninoculated one. Zhang et al. (2007) also provided similar evidence that PGPR can elicit changes in endogenous plant auxin homeostasis without providing microbial-originating auxin. In roots, the transcriptional level of IAA biosynthesis genes increases or remains unchanged upon inoculation. However, in primary and lateral root meristems, DR5::GUS transgenic plants revealed higher auxin levels, signifying that the tissue distribution of auxin or its polar transport is affected by the bacterium. Zhang et al. (2007) also reported that GB03 exposed DR5::GUS plants to *B. subtilis* led to the proposal that the PGPR strain not only affects auxin biosynthesis but also tissue homeostasis and polar transport.

Auxin as an auxin reflux carrier is supposed to be in *Arabidopsis*; auxin transport charged IAA to the cytoplasm (Péret et al., 2012). In rice, auxin is localized to the plasma membrane (Yu et al., 2015). Auxin-responsive genes in monocots include three families: Aux/IAAs, GH3s, and small auxin-up RNAs (SAURs) (Wang et al., 2010b). Previous studies also demonstrated that most of the group II GH3 subfamily members are IAA–amido synthetases that maintain endogenous auxin homeostasis by conjugating excess auxin with amino acids (Khan and Stone, 2007; Fu et al., 2011). Results showed that in rice, the GH3-2 gene localizes to the ER membrane. According to these findings, the interaction of the *OsGH3-2* gene with the ipdc gene showed positive signals in rice protoplast toward plant–microbe interaction; it can be assumed that the interaction of the *ipdc* gene with the GH3-2 synthetase gene influences IAA biosynthesis, resulting in enhanced LR and RHs.

Cd also affects the distribution and metabolism by interrupting auxin homeostasis in *Arabidopsis* (Hu et al., 2013). Cd sensitivity was increased by a reduction in auxin content. A study on *osaux1-3* mutant lines revealed that PR, LR, and HR are more sensitive to Cd than wild-type. The death rate of root cells becomes higher in *osaux1* mutants exposed to Cd stress, as determined by histochemical staining with Hoechst 33342 and propidium iodide. In the *osaux1* mutant, the Cd content was higher as compared to the wild-type, while upon inoculation with bacterial strain, the level of Cd content became lower under stress conditions. Results also demonstrated that the death rate of the bacterial cells was not significantly high or that Cd did not completely retard the growth rate of *B. cereus*, suggesting that *B. cereus* has a survival capability in response to Cd stress. Our findings are consistent with those of Sabater and Martín (2013); Souza et al. (2015), and Dubey et al. (2014), who reported that the production of reactive oxygen species causes plant cell death under stress conditions. Ecological stress generates several ROS. ${\text{O}}_{2}^{-}$ is a primary reactive oxygen species for toxic heavy metals and triggers the generation of OH˙ and O^2^ as ROS (Das and Roychoudhury, 2014). The H_2_O_2_ concentration in the wild-type increased significantly after 3 days of Cd exposure, similar to the findings of Krishnamurthy and Rathinasabapathi (2013) in *Arabidopsis* under Cd treatment. ${\text{O}}_{2}^{-}$ and H_2_O_2_ accumulation is the cause of oxidative stress. In *osaux1*, ${\text{O}}_{2}^{-}$ and H_2_O_2_ are expressed at higher levels than in the wild-type under stress conditions. Through reactive oxygen species-mediating signaling, it can be assumed that in rice, auxin transport through *osaux1-3* H_2_O_2_ has a regulating role during tolerance to Cd stress conditions. Previous studies have already reported that in *Arabidopsis*, under unfavorable conditions, cell cycle regulation and auxin signaling function regulate root development (De Smet et al., 2010; Olanrewaju et al., 2017). Despite the effect of *B. cereus* on IAA biosynthesis and transport, IAA genes showed downregulation in the transcript level of *osaux1-3* compared to wild-type.

## Conclusion

Our results indicate that a functional IAA signaling pathway is required for root architecture and lateral root development in response to *Bacillus cereus*. Although the action of PGPR on roots due to the involvement of auxin has long been said to originate from bacteria, it is the first key step of the auxin transduction pathway required in plant response. We showed that PGPR strains in the host plant affect the endogenous IAA homeostasis in a complex manner by interacting with the *GH3-2* gene without directly providing IAA. Nevertheless, the mechanism of ipdc, the precursor gene of IAA in bacterial strains, and GH3-2 in host–plant interaction is not clearly understood. Therefore, further research is required at the domain level to understand their mode of function and how they interact with each other in response to root development.

## Data availability statement

The original contributions presented in the study are included in the article/**Supplementary Material**. Further inquiries can be directed to the corresponding authors.

## Author contributions

TJ conceived of the research idea and supervised the research. GS conducted the research, collected data, and did a formal analysis of the data. MJ and GS wrote the original draft of the manuscript. SF, KA and NK provided technical expertise to strengthen the research concept. SF and SY helped in the funding acquisition. All authors contributed to the article and approved the submitted version.

## Funding

This work was carried out with the support of Cooperative Research Program for Agriculture Science and Technology Development (Project No. PJ017068032022) Rural Development Administration, Republic of Korea.

## Acknowledgments

The authors extend their appreciation to the Researchers Supporting Project number (RSP-2021/369), King Saud University, Riyadh, Saudi Arabia. We would like to thank Dr. Adeel Abbas, Dr. Rashida Hameed and Dr. Muhammad Sajid for their help and guidance for this work and reviewing of the manuscript.

## Conflict of interest

The authors declare that the research was conducted in the absence of any commercial or financial relationships that could be construed as a potential conflict of interest.

## Publisher’s note

All claims expressed in this article are solely those of the authors and do not necessarily represent those of their affiliated organizations, or those of the publisher, the editors and the reviewers. Any product that may be evaluated in this article, or claim that may be made by its manufacturer, is not guaranteed or endorsed by the publisher.

## Abbreviations

IpdC, indole-3-pyruvate decarboxylase; *GH3-2*, indole-3-acetic acid–amido synthetase gene; IPyA, indole-3-pyruvic acid; IAA, indole-3-acetic acid, Cd, cadmium.
