# Supplementary material for: Indole pyruvate decarboxylase gene regulates the auxin synthesis pathway in rice by interacting with the indole-3-acetic acid–amido synthetase gene, promoting root hair development under cadmium stress
Source: Front Plant Sci. 2022 Oct 21;13:1023723. doi: 10.3389/fpls.2022.1023723 (PMC9635337; doi:10.3389/fpls.2022.1023723)
Supplement: Supplementary file 1 [file Table_1.docx]

**Supplementary Table 1:** List of Primers used in plant-microbe interaction.

| **Name** | **Primer Sequence (3′to 5′)** |
| --- | --- |
| 35S:ORF_AUX1_-GFP U/L | GAGCTCATGGTGCCGCGCGAGCAGGC  GGATCCGTGGTGCGGCAATGGCACCG |
| AUX1qRT U/L | GCCTGCGCGAGTAACATCTA  CAGCACCAGCTTGGTTGGAC |
| O_S_ACTIN U/L | TCAGCAACTGGGATGATATGGAG  GCCGTTGTGGTGAATGACTAAC |
| Ngus RB | AACGCTGATCAATTCCACAG |
| IPDCqRT U/L | GGATCCCTTGAAAAAACAATAT  GGTTCAGCAAAATTCTGAGCTC |
| YUCCAqRT U/L | ATGGACCCTTGGAGTGAAAT  TACCTGGGAACCTCACTTTA |
| IPDC-GFP | TCATCATCATAGATCTATGAATTAGGAATTGAACATATATT  AGATCTGGTCAGCAAAATTCTAAGCTTGGTGGAGCTA |
| GH3-2 YFP | GGAGAGGACCTCGAGAGATCTATGTTGGAGAAGAAGGC  TTCAGCTCCGCCTACGACAAGCTTGGTGGAGCTGGTGGA |
|  |  |
